# Genetic properties of feed efficiency parameters in meat-type chickens

**DOI:** 10.1186/1297-9686-42-25

**Published:** 2010-06-29

**Authors:** Samuel E Aggrey, Arthur B Karnuah, Bram Sebastian, Nicholas B Anthony

**Affiliations:** 1 Department of Poultry Science, University of Georgia, Athens, GA 30602, USA; 2 Institute of Bioinformatics, University of Georgia, Athens, GA 30602, USA; 3 Department of Poultry Science, University of Arkansas, Fayetteville, AR 72701, USA

## Abstract

**Background:**

Feed cost constitutes about 70% of the cost of raising broilers, but the efficiency of feed utilization has not kept up the growth potential of today's broilers. Improvement in feed efficiency would reduce the amount of feed required for growth, the production cost and the amount of nitrogenous waste. We studied residual feed intake (RFI) and feed conversion ratio (FCR) over two age periods to delineate their genetic inter-relationships.

**Methods:**

We used an animal model combined with Gibb sampling to estimate genetic parameters in a pedigreed random mating broiler control population.

**Results:**

Heritability of RFI and FCR was 0.42-0.45. Thus selection on RFI was expected to improve feed efficiency and subsequently reduce feed intake (FI). Whereas the genetic correlation between RFI and body weight gain (BWG) at days 28-35 was moderately positive, it was negligible at days 35-42. Therefore, the timing of selection for RFI will influence the expected response. Selection for improved RFI at days 28-35 will reduce FI, but also increase growth rate. However, selection for improved RFI at days 35-42 will reduce FI without any significant change in growth rate. The nature of the pleiotropic relationship between RFI and FCR may be dependent on age, and consequently the molecular factors that govern RFI and FCR may also depend on stage of development, or on the nature of resource allocation of FI above maintenance directed towards protein accretion and fat deposition. The insignificant genetic correlation between RFI and BWG at days 35-42 demonstrates the independence of RFI on the level of production, thereby making it possible to study the molecular, physiological and nutrient digestibility mechanisms underlying RFI without the confounding effects of growth. The heritability estimate of FCR was 0.49 and 0.41 for days 28-35 and days 35-42, respectively.

**Conclusion:**

Selection for FCR will improve efficiency of feed utilization but because of the genetic dependence of FCR and its components, selection based on FCR will reduce FI and increase growth rate. However, the correlated responses in both FI and BWG cannot be predicted accurately because of the inherent problem of FCR being a ratio trait.

## Background

Feed cost constitutes about 70% of the total cost of live production, but the efficiency of feed utilization has not kept up the growth potential of today's broilers. Improvement in feed efficiency will reduce the amount of feed required for growth, the production cost and the amount of nitrogenous waste [1]. Efficiency in feed intake (FI) is more difficult to quantify than growth, and as a result different measures of feed efficiency have been developed, each of which reflects different mathematical and biological aspects of efficiency. In broiler chickens, feed efficiency is usually expressed as the amount of FI per body weight gain (BWG) referred to as feed conversion ratio (FCR). However, Chambers and Lin [2] have shown that a large proportion of the variation in FI and age constant FCR among broilers are due to body weight (BW) and efficiency of nutrient utilization. Also, variability in maintenance requirement, a major contributing factor to FI, is not accounted for in FCR. Statistically, FCR is a ratio trait and is not normally distributed, with no real mean and variance, and according to Atchley et al. [3] the non-normality of a ratio trait is increased when the magnitude of coefficient of variation of the denominator is increased. Pearson [4] has derived formulae to approximate the variance of a ratio and phenotypic correlation between two ratios but the lack of genetic independence of FCR from FI and BWG makes it difficult to improve without direct effect on growth.

Koch et al. [5] have introduced the concept of residual feed intake (RFI) that accounts for both maintenance requirements and growth. Residual FI represents the amount of FI not accounted for by maintenance BW and BWG. Selection on RFI has been proposed to improve feed efficiency because of its phenotypic independence of maintenance BW and BWG. The phenotypic independence of RFI from its estimating components is the direct result of the distributing properties of the regression procedure [6].

Kennedy et al. [7] have shown that genetic variability in RFI is not independent of metabolic BW and BWG. Luiting [8] have demonstrated that feeding behavior, nutrient digestibility, maintenance requirements, and energy homeostasis and partitioning affect RFI in laying hens. Aggrey et al. [9] have demonstrated that the proportion of protein energy retained is associated with feed efficiency. Jorgensen et al. [10] have also shown that variability in apparent metabolizable energy requirements affects feed efficiency in meat-type birds. Therefore, RFI may reflect more the variability in maintenance BW than differences in BWG. Genetic variability in RFI has been investigated in beef cattle [5,11-14] and pigs [15-17]. To date, there are only a few studies on RFI in broilers with heritabilities ranging from 0.21-0.49 [19,20]. Estimates on genetic correlation between RFI and BWG have ranged from almost zero [11,20] to positive values [17]. These differences may be due to sample size or statistical methods of estimation. The true genetic relationship between RFI and its components or lack thereof would allow for an accurate predictive correlated response to selecting for RFI.

The aim of the current study was to estimate genetic parameters pertaining to RFI and FCR of a growing broiler control population at two time periods and to ascertain the genetic relationships among the parameters that contribute to feed efficiency.

## Methods

### Population and animal husbandry

A pedigreed population was established from the Arkansas random bred population. Twenty-four males were pedigree mated to 72 females to produce 2,400 chicks in eight hatches. Chicks were sexed at hatching and placed in pens (0.074 m^2^/bird) with litter and fed a starter ration containing 225 g/kg protein, 52.8 g/kg fat, 25.3 g/kg fiber, 12.90 MJ ME/kg, 9.5 g/kg calcium (Ca), and 7.2 g/kg total phosphorus (P) (4.5 g/kg available P) until 18 days of age. Hereafter, they were fed a grower ration of 205 g/kg protein, 57.6 g/kg fat, 25.0 g/kg fiber, 13.20 MJ ME/kg, 9.0 g/kg Ca and 6.7 g/kg total P (4.1 g/kg available P). At 28 days, birds were fasted for 12 hours and transferred to individual metabolic cages (width = 20.32 cm; length = 60.96 cm; height = 30.48 cm) until 42 days of age. The birds were kept on an 20L:4D light regimen. BW was measured on days 28, 35 and 42. FI was measured at days 28-35 and 35-42. The metabolic body weights (BW^0.75^) at days 28 and 35 (MBW_28 _and MBW_35_) FI at days 28-35 and 35-42 (FI_28-35 _and FI_35-42_), FCR (FCR_28-35 _and FCR_35-42_) and residual RFI (RFI_28-35 _and RFI_35-42_) at days 28-35 and 35-42 were calculated. RFI was calculated as:

where a is the intercept and b_1 _and b_2 _are of partial regression coefficients of FI on BW^0.75 ^and BWG, respectively. Residual feed intake values were generated using regression procedure of SAS [18]. Experimental protocols were in accordance with the procedures of the University of Georgia institutional animal care and use committee.

### Data editing and analytical algorithm

After data editing, there were 2,289 animals with complete records and 104 (including eight grandsires) with no records. The animal model used to calculate heritability and genetic correlations of the traits is:

where Y_ijk _is the record of the k^th ^chicken from the i^th ^hatch and j^th ^sex; Hatch_i _= fixed effect of hatch (i = 1,...,8); Sex_j _= fixed effect of sex (j = 1, 2-male/female); a_k _= random direct additive genetic effect of individual k, and e_ijk _= random residual error. Analyses were performed using the GIBBS2F90 program based on a Markov Chain Monte Carlo approach. We assumed flat priors for systematic and random effects. The marginal posterior distribution of the trait of interest was obtained using Gibbs sampling. A single chain of 250,000-cycles length was generated. A burn-in period of 150,000 iterations was used as well as a 10-cycle lag to reduce autocorrelation among samples. A total of 10,000 samples were kept for post Gibbs analysis using the POSTGIBBSF90 program (with graph) [21] to compute the posterior means (point estimate for traits), and the 95% highest posterior regions (HPD95%) of heritability and genetic correlations of the traits. Convergence was ascertained by employing the algorithm of Raftery and Lewis [22]. Bivariate analyses were performed to compute genetic correlations between combinations of traits.

## Results

The means, standard deviations (SD) and heritabilities of the studied traits are presented in Table 1 and estimates of genetic correlation among feed efficiency parameters in Table 2. The heritability estimates of FCR_28-35 _and RFI_28-35 _were 0.49 and 0.45, respectively. Similarly, the heritability estimates for FCR and RFI at day 35 were 0.41 and 0.42, respectively. Whereas the genetic correlation between FCR_28-35 _and RFI_28-35 _was 0.31, the estimate between FCR_35-42 _and RFI_35-42 _was 0.84. The heritability estimates for MBW, BWG and FI for both periods were moderate ranging from 0.42 to 0.51. The genetic correlations between RFI_28-35 _and its components (MBW_28_, BWG_28-35 _and FI_28-35_) were all positive ranging from 0.29 to 0.56. However, the genetic correlation between RFI_35-42 _was 0.45 and 0.33 for MBW_35 _and FI_35-42_, respectively, but almost zero (0.06) for BWG_35-42_. The genetic correlations between the two RFI and FCR were 0.59 and 0.55, respectively. The RFI_28-35 _parameters were all moderately correlated (0.44-0.51) with RFI_35-42_, but the genetic relationship between BWG_35-42 _and RFI_28-35 _was almost zero (-0.05). The genetic correlation between FCR and MBW was moderately positive at both ages, and between FCR and BWG was slightly negative also at both ages. Feed efficiency parameters at days 28-35 were all positively correlated with FCR_35-42_, but the genetic correlation between BWG_35-42 _and FCR_28-35 _was almost zero (0.04). The genetic correlation between FCR_28-35 _and FCR_35-42 _was 0.55.

**Table 1:** Means (SD) and posterior means of heritability (95% highest posterior density region intervals) of feed efficiency parameters in meat-type chickens

**Trait**^**1,2**^	**Period**^**3**^	Means (SD)	Heritability
MBW	28	158.64 (17.27)	0.45 (0.44-0.46)
BWG	28-35	364.66 (88.04)	0.51 (0.50-0.52)
FI	28-35	655.28 (130.38)	0.48 (0.48-0.49)
FCR	28-35	1.84 (0.33)	0.49 (0.47-0.51)
RFI	28-35	0.00 (79.07)	0.45 (0.45-0.46)
MBW	35	206.74 (20.84)	0.49 (0.48-0.50)
BWG	35-42	455.58 (90.50)	0.48 (0.46-0.49)
FI	35-42	887.25 (146.35)	0.46 (0.46-0.48)
FCR	35-42	2.00 (0.42)	0.41 (0.41-0.43)
RFI		0.00 (114.07)	0.42 (0.42-0.43)

^1^MBW = Metabolic body weight (BW); BWG = BW gain; FI = feed intake; FCR = feed conversion ratio; RFI = residual feed intake

^2^All traits measured in (g) except for FCR which was (g/g)

^3^Age (d) or age range (d) that trait was measured

**Table 2:** Posterior means of genetic correlations (r_a_) (95% highest regions intervals) of residual feed intake (RFI) and feed conversion ratio (FCR) parameters in meat-type chickens

**Trait**^**1,2**^	**Period**^**3**^	**r**_**a**_**with RFI, 28-35 d**	**r**_**a**_**with RFI, 35-42 d**	**r**_**a**_**with FCR, 28-35d**	**r**_**a**_**with FCR, 35-42 d**
MBW	28	0.29 (0.29-0.31)	0.49 (0.48-0.49)	0.62 (0.61-0.62)	0.55 (0.55-0.56)
BWG	28-35	0.34 (0.33-0.35)	0.44 (0.43-0.44)	-0.13 (-0.13- -0.12)	0.56 (0.56-0.57)
FI	28-35	0.56 (0.55-0.57)	0.51 (0.51-0.52)	0.45 (0.44-0.46)	0.58 (0.57-0.58)
FCR	28-35	0.31 (0.30-0.31)	0.61 (0.61-0.62)		0.55 (0.54-0.56)
RFI	28-35		0.59 (0.58-0.59)		
MBW	35	0.31 (0.30-0.33)	0.45 (0.44-0.46)	0.32 (0.32-0.32)	0.57 (0.56-0.58)
BWG	35-42	-0.05 (-0.06- -0.04)	0.06 (0.05-0.07)	0.04 (0.02-0.05)	-0.14 (-0.14- -0.14)
FI	35-42	0.22 (0.21-0.23)	0.33 (0.33-0.34)	0.53 (0.52-0.54)	0.54 (0.54-0.55)
FCR	35-42	0.56 (0.55-0.56)	0.84 (0.84-0.85)		

^1^MBW = Metabolic body weight (BW); BWG = BW gain; FI = feed intake; FCR = feed conversion ratio; RFI = residual feed intake

^2^All traits measured in (g) except for FCR which was (g/g)

^3^Age (d) or age range (d) that trait was measured

## Discussion

The heritability estimates for both FCR and RFI for both periods were higher than the estimate of Van Bebber and Mercer [19], however, they were within the limits of published data in beef cattle and pigs [11,12,16,17]. Based on the genetic parameter estimates, selection for low RFI will improve feed efficiency with an expected correlated response in reduced FI. This will also favor birds with lower maintenance energy requirements based on the genetic correlation between RFI and MBW. However, there is a genetic dependency between RFI_28-35 _and BWG_28-35_. The positive genetic correlation between RFI_28-35 _and BWG_28-35 _suggests that fast growing chickens have greater appetite and consume more feed than needed for growth. This dependency does not exist at days 35-42. Therefore, selection at days 35-42 may be more attractive than at days 28-35. Feed efficiency is a compound trait affected by both feed- and growth-related factors, and these factors vary with age. Therefore the genetic relationships among feed efficiency parameters are also expected to vary with age.

In the current study, the genetic interrelationships among the feed efficiency parameters were different at days 28-35 and days 35-42. The lack of genetic correlation between RFI_35-42 _and BWG_35-42 _was similar to that reported in cattle and pigs [11,12,14,16,23]. However, Cai et al. [17] and Hogue et al. [24] have also reported a positive genetic correlation between RFI and average daily gain (ADG) in pigs selected for low RFI, which is similar to the genetic correlation between RFI_28-35 _and BWG_28-35 _obtained in this study. The change in genetic correlation between RFI and BWG with age could be due to differences in body composition during the two periods when RFI was determined. Jensen et al. [25] have also obtained genetic correlations between RFI and ADG of 0.32 and -0.24 at two different ages. In pigs, RFI is negatively correlated to dressing percentage and positively correlated with backfat thickness [16]. The body composition of broiler chickens at days 28-35 is different from that of at days 35-42, therefore it is possible that the internal allocation of resources above maintenance into protein accretion and fat deposition among others could contribute towards the different inter-relationships between factors that affect RFI at these two time periods.

Feed efficiency measured over a long period of time is possibly an aggregate efficiency over different developmental processes which can vary from species to species as well as the management practices under which animals are raised. In meat-type birds, feather development, feeding behavior, skeletal growth, tissue accretion and fat deposition are different developmental processes all of which or combinations of which can affect heritability of RFI and also the genetic correlations among RFI parameters.

In the literature on broilers while data on RFI is scant, information on FCR is abundant possibly due to its ease of computation and to the producers' direct association of cost and profits to quantities of feed. The heritability estimate of FCR was 0.49 and 0.41 for days 28-35 and days 35-42, respectively. FCR is a ratio trait that is not normally distributed [26] and is subject to skewness and kurtosis as a result of the changes in BWG (denominator) coefficient of variation and subsequently affect SD, covariances and correlations [3]. Selection for FCR will improve efficiency of feed utilization but because of the genetic dependence of FCR and its components, selection for reduced FCR will reduce FI and increase growth rate. Increases in both FI and BWG cannot be predicted accurately because of the inherent problem of FCR being a ratio trait. Lin [27] has developed a linear index based on the components of FCR. Gunsett [28], Famula [29] and Campo and Rodriguez [30] have shown that the linear index is more efficient than direct selection on the ratio. However, Gunsett [28] has also pointed out that the advantage of the linear index decreases as the correlation between the two component traits increases or as the heritability of both components moves towards equality.

The genetic correlation between RFI and FCR was 0.31 at days 28-35 compared to 0.84 at days 35-42. This suggests that the nature of the pleiotropic relationship between RFI and FCR may be dependent on age, and consequently the molecular, physiological and nutritional factors that govern RFI and FCR may also depend on time of development, or on the nature of resource allocation of FI above maintenance designated for protein accretion and fat deposition. The lack of genetic correlation between RFI and BWG at days 35-42 provides the independence of RFI on the level of production, thereby making it possible to study the molecular, physiological and nutrient digestibility mechanisms underlying RFI without the confounding effects of growth.

Estimating genetic properties of RFI provides the genetic parameters that are needed in combination with economic values in the selection criteria in order to ascertain the economic benefits of selecting for feed efficiency.

## Competing interests

The authors declare that they have no competing interests.

## Authors' contributions

SEA designed the experiment, analyzed the data and drafted the manuscript. NBA was responsible for breeding the animals. ABK and BS assisted in execution of the experiment. All authors submitted comments, and read and approved the final manuscript.
